# Bioimpedance Sensing of Implanted Stent Occlusions: Smart Stent

**DOI:** 10.3390/bios12060416

**Published:** 2022-06-15

**Authors:** Antonio Rodríguez, Pablo Barroso, Alberto Olmo, Alberto Yúfera

**Affiliations:** 1 Instituto de Microelectrónica de Sevilla (IMSE-CSIC), Universidad de Sevilla, 41012 Sevilla, Spain; antoniorodriarjo@gmail.com (A.R.); yufera@us.es (A.Y.); 2 Departamento de Física Aplicada III, Universidad de Sevilla, 41012 Sevilla, Spain; pablobarroso@us.es

**Keywords:** Coronary artery disease, restenosis, stent, bioimpedance measurement

## Abstract

Coronary artery disease is one of the most common diseases in developed countries and affects a large part of the population of developing countries. Preventing restenosis in patients with implanted stents is an important current medical problem. The purpose of this work is to analyse the viability of bioimpedance sensing to detect the formation of atheromatous plaque in an implantable stent. Simulations in COMSOL Multiphysics were performed to analyse the performance of the proposed bioimpedance sensing system, based on the Sheffield technique. Both non-pathological and pathological models (with atheromatous plaque), including the flow of blood were considered. Simulations with the non-pathological model showed a homogeneous distribution of the measured current intensity in the different electrodes, for every configuration. On the other hand, simulations with the pathological model showed a significant decrease of the measured current intensity in the electrodes close to the simulated atheromatous plaque. The presence of the atheromatous plaque can, therefore, be detected by the system with a simple algorithm, avoiding the full reconstruction of the image and the subsequent computational processing requirements.

## 1. Introduction

Coronary artery disease (CAD) has been the leading cause of mortality worldwide for decades [1]. Coronary artery disease, also called ischemic heart disease (IHD), involves the reduction of blood flow to the heart muscle. The most common cause of CAD is coronary artery stenosis [1], which is a narrowing of the coronary lumen space caused by an atherosclerotic lesion [2]. One of the main treatments for coronary artery stenosis, as well as for coronary artery disease in general, consists of the implantation of a stent that returns the diameter of the vessel lumen to the approximate size of the original healthy vessel. The introduction of coronary stents marked a major turning point in the practice of interventional cardiology [3].

In-stent restenosis (ISR), the narrowing of a stented coronary artery lesion, has been in the past an important medical problem. However, technological advances and an improved understanding of the restenotic process have improved the overall rate of in-stent restenosis following stent implantation [3].

Different approaches have been followed to prevent ISR. Drug-eluting stents (DESs) significantly reduce the incidence of restenosis and improve patient prognosis [4]. The use of drug-eluting stents has important implications for the care of individual patients and the delivery and economics of health care in general [5]. Different drugs are being tested as a potential therapeutic approach for ameliorating vascular occlusive diseases [6]. Diabetes, multiple stents, and smaller final minimal lumen diameter (MLD) are strong predictors of restenosis after coronary stent placement [7]. Furthermore, recent advancement of non-invasive coronary computed tomography (CT) allows detection of in-stent restenosis with great sensitivity and specificity [8,9].

As a different approach, the use of an intelligent stent to monitor restenosis has been recently proposed [10], to achieve a real-time monitoring of the stent and avoid CT sessions. This device would be integrated with the stent and would be able to collect data remotely, so it would not require further invasive techniques. An initial mathematical model was developed to analytically describe the histological composition of the neointima, employing its conductivity and permittivity data [10]. In another work [11], a 4-electrode setup for bioimpedance measurements was proposed. This setup allowed the study of the influence of the various tissues involved in restenosis: fat, muscle, fibre, and endothelium, together with the blood, with interesting and promising results for the local monitoring of restenosis with bioimpedance measurements.

In this work, we have analysed a complete artery-stent system. Two models have been studied: a non-pathological model, including the flow of blood, and a pathological model, with a characteristic formation of atheromatous plaque. An Electrical Impedance Tomography system, based on the Sheffield technique, is proposed, with the use of eight electrodes surrounding the stent circumference. We used COMSOL Multiphysics for the finite element simulations carried out. The AC/DC and Laminar Flow Analysis modules were used for the presented simulations. Different practical remaining challenges in the fabrication of intelligent stents are addressed, such as the miniaturization of the technology, the biocompatibility of materials used or the need for clinical security.

## 2. Materials and Methods

Section 2.1 describes the artery model used, its limitations and improvements over previous models. Section 3.1 describes the impedance system used, together with the parameters of our simulations.

### 2.1. Artery Model

There are three types of arteries: elastic, muscular and arterioles. Elastic arteries have a higher proportion of elastin in their walls [12]. The walls of muscular arteries, also called distribution arteries, consist of smooth muscle fibres arranged in a spiral, that regulate the size of the vessel and the amount of blood circulating during a given time interval. Finally, the arterioles are smaller, and have the function of lowering the blood pressure so that the capillaries, whose walls are extremely thin, are not damaged. In our work, we focused on the coronary arteries, which can be understood as a type of muscular artery with certain peculiarities in its intimate layer, keeping more similarities with those of the elastic arteries than with those of the muscular ones [12].

**Figure 1:**
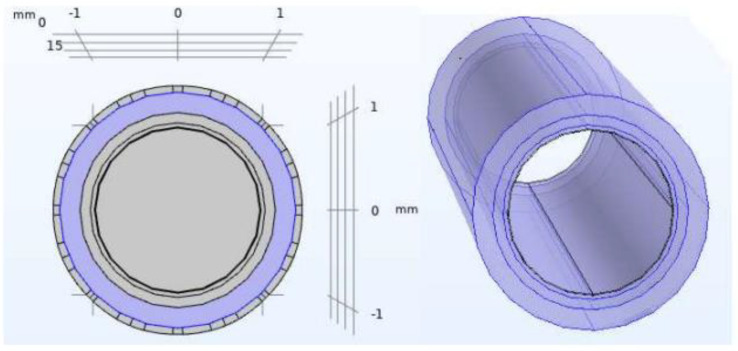
Non−pathological artery model. Geometry of the different layers included in the COMSOL model: blood, endothelium, fibre layer, fat layer (from the inner layer to outer layer).

For the non-pathological model of the artery, our model targets the moment when the neointima is completed homogeneously throughout the internal contour of the vessel, and just before the stenosis starts. These layers are, from the outside towards the lumen of the artery, the layer of fat, muscle, fibre and endothelium, finally ending up in an inner cylinder of blood. The geometry of the model is shown in Figure 1. The values used to implement that geometry, and the electrical properties of the different layers were extracted from [11] and are summarized in Table 1, Table 2 and Table 3.

**Figure 2:**
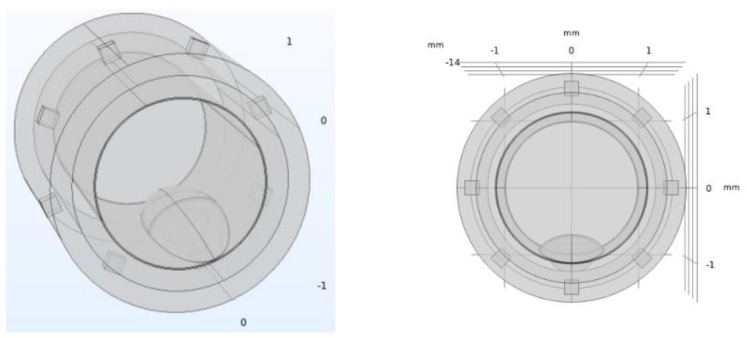
Pathological artery model with atheromatous plaque. The atheromatous plaque is represented at the bottom part of the inner circumference, and it is built from the intersection of different ellipsoids. Eight electrodes for the electrical impedance tomography stem are also represented, surrounding the diameter of the stent, as explained in Section 2.2.

For the pathological model of the artery, the atheromatous plaque was characterized as a thickening in the muscular layer (where the atherocytes would be found), although with high liposuction content, which pushes the endothelial layer towards the light. In this way, this plaque will have the same electrical properties (permittivity and conductivity) than those assigned to the fat layer. Figure 2 shows the geometry used for the pathological model of the artery, including the atheromatous plaque modelled. The intra luminal ellipsoid shape is well in line with the in-stent calcified nodules described in [13].

**Figure 3:**
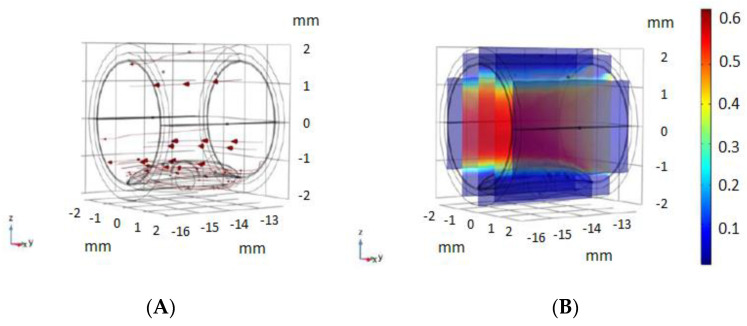
Blood movement model used. (**A**) blood flow movement vectors, using the Laminar Flow Analysis module of COMSOL. (**B)** Velocity magnitude of blood flow (m/s) used in our model, with the conditions used in different models [14,15].

Simulations were carried out considering the movement of the blood, as shown in Figure 3. The conditions for the blood fluid model were obtained following different models used in [14,15], using the Laminar Flow Analysis module of COMSOL Multiphysics with the dimensions of the artery previously shown, and the flow parameters shown in Figure 3. The movement of the blood is an important parameter for the monitoring of impedance, and its simulation improves the model reported in [11].

### 2.2. Electrical Impedance Tomography System Model and Simulations Performed

Electrical Impedance Tomography (EIT) is a non-invasive diagnostic imaging method, where the impedance of a biological tissue or part of the body is inferred from surface electrode measurements. The EIT method has long been used in biomedical engineering for cancer detection [16], respiratory problems monitoring [17] or cardiac imaging [18], among other applications. This method uses low-frequency electrical current applied through surface electrodes to determine differences in electrical conductivity (or impedance) of biological regions through the body. There are different configurations for EIT [19], and different algorithms can be used for image reconstruction [20].

The selected method to carry out the Electrical Impedance Tomography (EIT) simulations was the Sheffield method. This method consists of the injection of current for each one of the surface electrodes, and measurements are obtained from the rest of the electrodes. This configuration is optimal for the detection of nearby anomalies to the contour of the region to be monitored [21].

**Figure 4:**
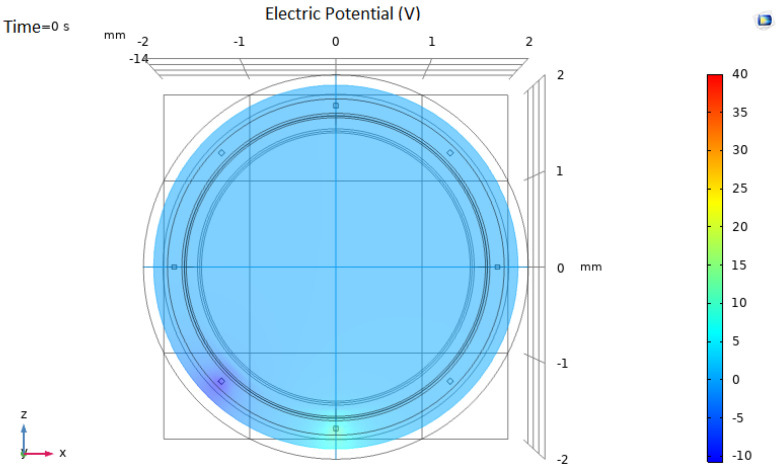
Electrical Impedance Tomography system model. In this particular setting (configuration 5), the lower electrode is set to 10 V and the contiguous electrode is set to −10 V. Current intensity is measured in the remaining 6 electrodes.

In our EIT model, eight gold electrodes (50 µm of size) were used in a circular shape, as shown in Figure 4. The electrodes were limited within the intimal layer, without exceeding the medial smooth muscle layer. Different settings were simulated. In each setting, two electrodes were excited at a determined voltage, and current intensity was measured in the remaining 6 electrodes. For setting *i,* electrode *i* was excited at +10 V, and *i* + 1 was set to −10 V. The current intensity was read from each one of the remaining electrodes.

As there are eight electrodes in our system, by changing the excitation and ground electrodes a total number of eight configurations were obtained. All the simulations were carried out on the permanent regime, at an excitation frequency of 10 kHz, with the corresponding values of conductivity and permittivity indicated in Table 1. The value of 10 kHz was selected as it was an intermediate value in Table 2 and Table 3. An initial voltage value of ±10 V was used for the excitation electrodes. This value was selected as it could be a practical value to use in our medical implants, although other values could also be used.

## 3. Results

The results section is divided in two subsections: Section 3.1, showing the simulation results for the non-pathological model, and Section 3.2, showing the simulation results for the pathological model.

### 3.1. Non-Pathological Model Simulations

**Figure 5:**
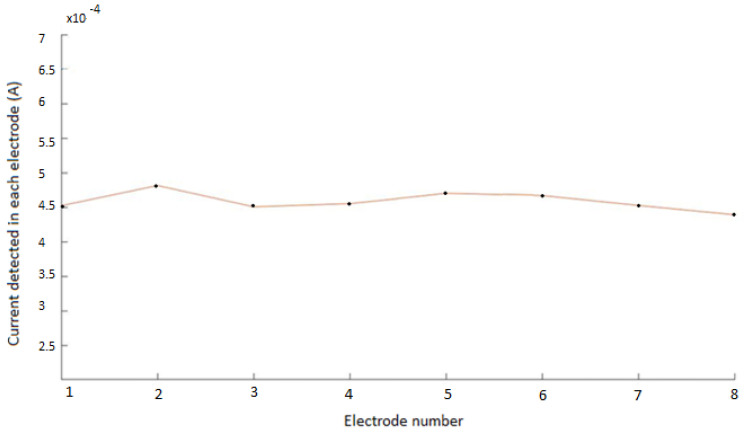
Electrical current measured in each electrode for the non−pathological model. The flat curve corresponds to the symmetry of the modelled system. Small variations (± 0.3 A) are due to the precision in the mesh used in COMSOL.

Simulations for the non-pathological model showed, as expected, a uniform and homogeneous distribution of the measured current intensity in the different electrodes, for every configuration. The electrical current measured in each electrode, for a specific configuration (configuration 1, with the upper electrode at +10 V), is shown in Figure 5. The electrical current measured was close to 4.5 A. The uniform measured values correspond well with the symmetry of the non-pathological model. Small variations (±0.3 A) are due to the error introduced by COMSOL in the mesh because of the precision in the simulation parameters, as can also be seen in Figure 5. Simulations were similar for the other seven configurations, showing in all cases the homogeneity of the model.

### 3.2. Pathological Model Simulations

**Figure 6:**
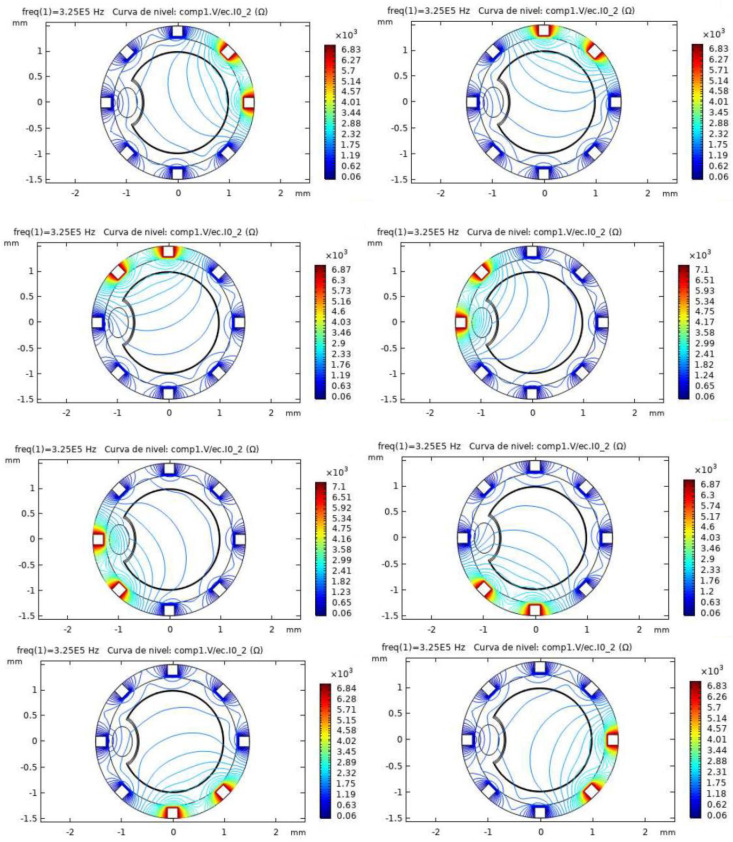
Simulation results for the pathological model in different configurations. The level curves for Impedance (ohms) obtained in COMSOL are shown. The atheroma generates a disturbance in the electric fields, as it can be seen near the left electrode for the different configurations.

Figure 6 shows the simulation results for the pathological model and different electrode configurations. The impedance field curves obtained for the eight different configurations are shown. It can be observed in the COMSOL simulations how a disturbance is generated in the impedance field level curves, breaking the previous symmetry. This disturbance corresponds to the presence of the additional material forming the atheroma.

**Figure 7:**
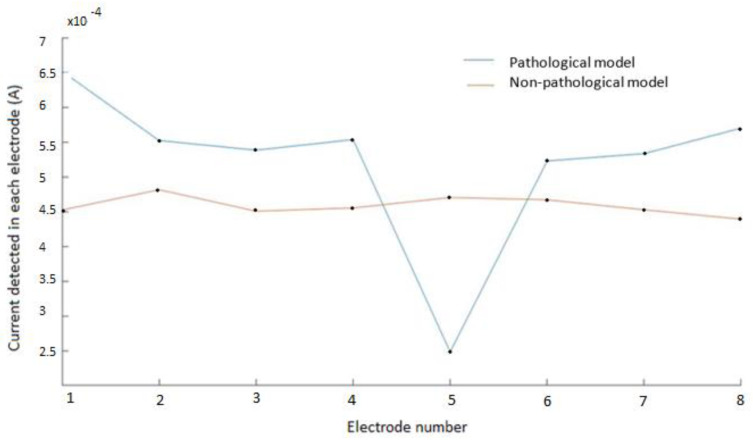
Electrical current measured in each electrode for the pathological model and the non−pathological model. Electrodes 1 and 2 represent the excitation electrodes, and electrode 5 is the electrode close to the modelled atheromatous plaque, as in Figure 2. The drop in the measured voltage for electrode 5 corresponds well with the presence of the atheromatous plaque. Similar results are obtained for other configurations.

Figure 7 shows the electrical current measured in each electrode for the pathological model, in one of the configurations, compared with the previous measurements in the non-pathological model (compare to Figure 5). As can be seen in the pathological model, there was a relevant decrease of the measured current intensity (above 50%) in the electrodes close to the simulated atheromatous plaque.

In the non-pathological model, the electrical current was approximately constant, only having the irregularities and inaccuracies of the model. The graph of the model with the atheroma shows a noticeable drop in the measured current in the electrode number 5 (close to the atheroma) with respect to its contiguous ones. Similar results were obtained for other configurations.

The high sensitivity of the measurements achieved is well in line with the expected results using the Sheffield method for EIT described in Section 2.2. Other methods commonly used in EIT are the Opposite method or the Cross method [22], which achieves a lower sensitivity in the limits (of utmost importance in the present application), or the trigonometric method [22], which requires a more complex technological implementation.

The relationship between the current detected in each electrode can be useful for determining the presence of atheromatous plaque, without the need to reconstruct the tomography image, and therefore reducing complexity in the overall process.

## 4. Discussion

The discussion is divided into two subsections: Section 4.1, which summarises the results of the simulations obtained and compares them with similar works, and Section 4.2, which summarises practical considerations for the future implementation of the proposed technique.

### 4.1. Use of Bioimpedance for the Monitoring of Stent Occlusions

The simulations carried out showed a uniform and homogenous distribution of the electric current for the non-pathological model. This is in accordance with previous partial results published in [11], where a mathematical model was developed to analytically describe the histological composition of the neointima, employing its conductivity and permittivity data.

In our complete artery-stent system, including the blood flow, we also studied a pathological model. The atheromatous plaque was characterized as a thickening in the muscular layer, modelled as the intersection of different ellipsoids. The selected method to carry out the Electrical Impedance Tomography (EIT) simulations was the Sheffield method, consisting of the injection of current in one of the surface electrodes and obtaining measurements from the rest of the eight electrodes, as in [21]. The results showed a relevant decrease of the measured current intensity in the electrodes close to the simulated atheromatous plaque.

The sensitivity of the measurements is well in line with the previous sensitivity reported for the Sheffield method [22]. Other methods commonly used in EIT are the Opposite method, the Cross method, or the trigonometric method. The Opposite method applies current through electrodes that are 180° apart, while voltage differences are measured on the remaining electrodes [22]. This method has a lower sensitivity in the limits of the circle, which is of utmost importance for the present application. The Cross method [23], where adjacent electrodes are selected as current and voltage references, also reaches higher sensitivity in internal regions. Gisser et al. [24] proposed a current injection method called the adaptive method or trigonometric method. In this method, current is injected on all electrodes and voltages are measured on all electrodes, and a good sensitivity is obtained, although the complexity of the system could have negative effects on the practical implementation of the medical implant.

The sensitivity of the data obtained can be seen as an important advantage for the simple and real-time monitoring of the evolution of the stent, being able with bioimpedance monitoring to detect the presence of the occlusion, without the need to implement EIT image reconstruction algorithms. The image reconstruction involves a mathematical inverse problem, for which accurate resolution demands large computation time and capacity. Even though different interesting current approaches based on Machine learning are being studied to improve calculation time [25], we think our approach could be more easily applied for this specific application, with sensitivity enough to provide an efficient clinical support.

The smart stent presented in this work would allow the real-time monitoring of potential ISR situations, avoiding for the patient the need to attend computed tomography sessions, or being applied in special situations, such as patients with mobility restrictions or allergies to DESs.

### 4.2. Practical Considerations and Future Work for the Implementation of the Smart Stent

There are important remaining challenges for the practical implementation of the smart stent. It will be necessary to study the implementation of the power supply for the intelligent stent, its integration and its fabrication in reduced dimensions and biocompatible materials. It will also be essential to study the clinical security, the placement of the integrated circuits in appropriated biological sites in order not to create additional stenosis problems due to the electronic components, in line with the general tendency of minimizing invasiveness [6]. Biocompatibility of the materials used is also essential, as other materials will be needed for the electronic development of the impedance measurement system, apart from traditional stent materials (cobalt chromium, 316 L stainless steel). A complete study of the clinical security of the different systems (power management system, wireless transmission modules, etc.) would need to be carried out. Stretchable electronics can be used for the fabrication of the intelligent stent and the required electrodes. Flexible and stretchable electronics are currently being proposed for a wide variety of biomedical implant applications. As recently proposed [26], stretchable electronics can be used itself as another way of measuring the diameter of the vessel lumen. In any case, the use of flexible electronics facilitates its use in implantable systems. Other works focus on low-cost diagnostic technology capable of monitoring multiple disease biomarkers in parallel [27] and new technologies to be used in optoelectronic systems [28]. The review of new technologies for the miniaturization of the device and the optimization of data processing will also be interesting in the future implementation of the medical implant. The proposed smart stent must fulfil the definition of a Class III medical device for therapeutic purposes, with an implantable long-term use, according to Regulation (EU) 2017/745 of the European Parliament.

Variability of bioimpedance among individuals should be considered. According to the data published in [11] and the present simulations performed, we consider the proposed device could be sensitive enough to detect occlusions formed inside the stent. However, other type of occlusions may exist, as shown in [3], such as occlusions taking place in an internal inner layer, that could lead to the bending of the existing stent. In that case the change in impedance should also be studied, and experimental models would be of interest. It will be essential to conduct simulations with more realistic clinical models, modelling the different necessary ISR cases.

## 5. Conclusions

The use of an intelligent stent to monitor restenosis has recently been proposed. In this work we modelled and simulated a complete artery-stent system. Two models were studied: a non-pathological model, including the flow of blood, and a pathological model, with a characteristic formation of atheromatous plaque. An Electrical Impedance Tomography system, based on the Sheffield technique, is proposed, with the use of eight electrodes surrounding the stent circumference. Simulations for the non-pathological model showed a uniform and homogenous distribution of the electric current. For the pathological model, there was a relevant decrease of the measured current intensity in the electrodes close to the simulated atheromatous plaque, showing the potential applicability of the technique. The proposed method for the detection of atheromatous plaque in stents avoids the need for image reconstruction, and therefore avoids the need to implement computational cost-effective EIT reconstruction algorithms.

## Figures and Tables

**Table 1:** Geometrical properties of the different layers modelled.

Geometrical Properties	
Blood Cylinder, Inner Radius	155.4 × 10^−5^ m
Endothelium layer, outer radius	157.5 × 10^−5^ m
Fibre layer, outer radius	160 × 10^−5^ m
Fat Layer, outer radius	200 × 10^−5^ m
Cylinder length	1600 × 10^−5^ m
Electrode side	5 × 10^−5^ m

**Table 2:** Electrical properties of the different layers modelled. Electrical conductivity values and electric permittivity values used in our model, at different frequencies [11].

	Electrical Conductivity (S/m)			
Frequency (Hz)	Fat	Muscle	Fibre	Endothelium
10^0^	0.035	0.2	0.25	0.05
10^1^	0.038	0.202	0.25	0.05
10^2^	0.041	0.267	0.25	0.05
10^3^	0.042	0.321	0.25	0.05
10^4^	0.043	0.341	0.25	0.05
10^5^	0.043	0.362	0.3	0.050
10^6^	0.044	0.503	0.350	0.146

**Table 3:** Electric permittivity values used in our model, at different frequencies [11].

	Electric relative Permittivity (ε_r_)			
Frequency (Hz)	Fat	Muscle	Fibre	Endothelium
10^0^	9.91 × 10^6^	2.62 × 10^7^	2.0 × 10^4^	3.61 × 10^3^
10^1^	5.03 × 10^6^	2.57 × 10^7^	2.0 × 10^4^	3.61 × 10^3^
10^2^	1.52 × 10^5^	9.33 × 10^6^	1.0 × 10^4^	3.61 × 10^3^
10^3^	1.93 × 10^4^	4.35 × 10^5^	1.0 × 10^3^	3.61 × 10^3^
10^4^	9.12 × 10^2^	2.59 × 10^4^	1.0 × 10^2^	3.61 × 10^3^
10^5^	1.01 × 10^2^	8.09 × 10^3^	2.0 × 10^1^	3.58 × 10^3^
10^6^	50.8	1.84 × 10^3^	10.0	1.85 × 10^3^

## Data Availability

Not applicable.

## Abbreviations

CAD	Coronary Artery Disease
ISR	in-stent restenosis
IHD	Ischemic Heart Disease
MLD	Minimal Lumen Diameter
DES	Drug-Eluting Stent
EIT	Electrical Impedance Tomography
